# Maternal death inquiry and response in India - the impact of contextual factors on defining an optimal model to help meet critical maternal health policy objectives

**DOI:** 10.1186/1478-4505-9-41

**Published:** 2011-11-30

**Authors:** Henry D Kalter, Pavitra Mohan, Archana Mishra, Narayan Gaonkar, Akhil B Biswas, Sudha Balakrishnan, Gaurav Arya, Marzio Babille

**Affiliations:** 1 Department of International Health, Johns Hopkins University Bloomberg School of Public Health, 615 North Wolfe Street, Baltimore, MD, 21205, USA; 2 UNICEF, B-9 Bhawani Singh Lane, C-Scheme, Opp. Nehru Sahkar Bhawan, Jaipur 302 001, Rajasthan, India; 3 Maternal Health, Directorate of Health Services, 3rd floor, Bank of India Building, Arera Hills, Bhopal, Madhya Pradesh, India; 4 UNICEF, E-7/650 Arera Colony, Shahpura, Bhopal 462 016, Madhya Pradesh, India; 5 Department of Community Medicine, RG Kar Medical College and Hospital, Kolkata, India; 6 UNICEF, 219/2, AJC Bose Road, Kolkata 700 017, West Bengal, India; 7 UNICEF, 73 Lodhi Estate, New Delhi 110 003, India

**Keywords:** Maternal mortality, health policy, verbal autopsy, death inquiry, community participation

## Abstract

**Background:**

Maternal death reviews have been utilized in several countries as a means of identifying social and health care quality issues affecting maternal survival. From 2005 to 2009, a standardized community-based maternal death inquiry and response initiative was implemented in eight Indian states with the aim of addressing critical maternal health policy objectives. However, state-specific contextual factors strongly influenced the effort's success. This paper examines the impact and implications of the contextual factors.

**Methods:**

We identified community, public health systems and governance related contextual factors thought to affect the implementation, utilization and up-scaling of the death inquiry process. Then, according to selected indicators, we documented the contextual factors' presence and their impact on the process' success in helping meet critical maternal health policy objectives in four districts of Rajasthan, Madhya Pradesh and West Bengal. Based on this assessment, we propose an optimal model for conducting community-based maternal death inquiries in India and similar settings.

**Results:**

The death inquiry process led to increases in maternal death notification and investigation whether civil society or government took charge of these tasks, stimulated sharing of the findings in multiple settings and contributed to the development of numerous evidence-based local, district and statewide maternal health interventions. NGO inputs were essential where communities, public health systems and governance were weak and boosted effectiveness in stronger settings. Public health systems participation was enabled by responsive and accountable governance. Communities participated most successfully through India's established local governance Panchayat Raj Institutions. In one instance this led to the development of a multi-faceted intervention well-integrated at multiple levels.

**Conclusions:**

The impact of several contextual factors on the death inquiry process could be discerned, and suggested an optimal implementation model. District and state government must mandate and support the process, while the district health office should provide overall coordination, manage the death inquiry data as part of its routine surveillance programme, and organize a highly participatory means, preferably within an existing structure, of sharing the findings with the community and developing evidence-based maternal health interventions. NGO assistance and the support of a development partner may be needed, particularly in locales with weaker communities, public health systems or governance.

## Background

Globally, in 2008 there were approximately 358, 000 maternal deaths, 99% of which occurred in low-income countries. The maternal mortality ratio (MMR) in India was estimated at 230 maternal deaths per 100, 000 live births, translating to about 63, 000 deaths or about 18% of the global total [1]. Obstetric complications are a leading cause of death among women of reproductive age, and for every woman who dies approximately 20 more suffer injuries, infection and disabilities related to pregnancy or childbirth [2].

The Indian public health system and programmes are currently undergoing a major reform. Following the recommendations of the International Conference on Population and Development, the Government of India is reorienting the family planning and maternal and child health programme into the new Reproductive and Child Health (RCH) Programme [3-5]. The programme's second phase, RCH-II, was initiated in 2005 and is grounded in the existing national health and population policies of the Tenth Five Year Plan [6-8]. Improving maternal health is a core goal, and reducing maternal mortality is a key objective to be achieved by increasing the number of facilities offering "safe delivery, emergency obstetric care and demand for these services" [9] in line with the principle that "every pregnant woman is at risk for life-threatening complications, and safe delivery and access to emergency obstetric care are essential" [10].

In 2005 the Government of India launched the National Rural Health Mission (NRHM), subsuming RCH-II. NRHM focuses on 18 states where health indicators are weakest, with the goal of providing accessible, affordable and accountable quality health services even to the poorest households in the remotest rural regions [11]. Maternal mortality reduction is a core priority, with an objective to reduce the MMR to below 100 by 2012.

Key NRHM strategies include upgrading 2, 000 community health centers (CHC) to first referral units (FRU) capable of providing emergency obstetric and neonatal care services, and fully operationalizing all CHCs and 50% of primary health care centers to provide round-the-clock delivery services and newborn care. NRHM also envisages empowering non-specialist medical officers to undertake emergency obstetric procedures and administer anesthesia; promoting delivery by skilled birth attendants; strengthening the skills of auxiliary nurse midwives to provide life-saving care during delivery; and stimulating demand for safe delivery through the Janani Suraksha Yojana (JSY) incentive scheme. JSY provides cash assistance for institutional antenatal, delivery and postnatal care coordinated with community care by a health worker.

However formidable the challenges of implementing these health system reforms, in the Indian context likely they are insufficient by themselves to overcome maternal mortality. Rural communities, with most of the population and higher mortality [12], are steeped in traditional beliefs and practices. People are unaware of danger signs indicating the need for urgent care in pregnancy and childbirth or where best to seek care, with husbands sometimes neglecting to take their wife due to costs or other priorities. Travel to the nearest health facility is often a distant ride over rough roads, if transport can be found and payment arranged. And health system reforms can be slow in achieving meaningful coverage, still leaving many women far from effective services at an appropriate facility. To help tackle these problems, conceptualized in the maternal health literature as the "three delays" [13], RCH-II and NRHM adopted priority strategies including promoting community participation in evidence-based health programme planning, with the objective of strengthening community-health system linkages and increasing community demand for quality, responsive RCH services.

**Figure 1:**
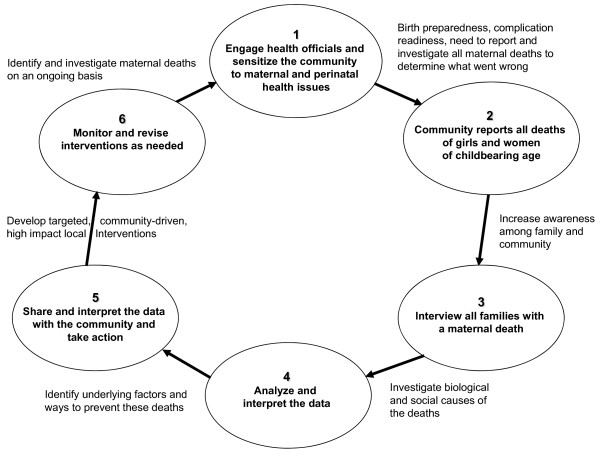
**MAPEDIR's six-step process**.

In the National RCH-II programme, maternal and infant death audit was considered an important example of reform and innovation. In select districts of India, the Maternal and Perinatal Death Inquiry and Response (MAPEDIR) process was devised to help activate this plan, bringing maternal deaths to light and providing actionable data to empower communities and inform local health systems. Vital statistics in India underestimate maternal mortality due to a high proportion of births and deaths occurring at home [14] as well as limited data on maternal deaths at health facilities [15,16]. Although villagers know of the maternal deaths in their midst, often they do not understand the importance of reporting these to the health system. Health workers who become aware of maternal deaths may not report them for fear they will be blamed. Survey data are valid only at the state and national levels [12], and do not include data on contributory social factors nor a means of sharing the data with communities. MAPEDIR, similar in ways to maternal death reviews in other developing countries [17-20], utilizes a verbal autopsy tool to conduct community-based confidential inquiries of recent maternal deaths to gather information on their causes and contributors. MAPEDIR goes a step further by encouraging the development of a community-health system partnership to build community and institutional awareness of maternal mortality, conduct and interpret the findings of the death inquiries, support the development of local data-driven interventions, and promote advocacy for and increased utilization of high quality maternal health care services. Figure 1 illustrates MAPEDIR's six-step process.

From 2005 to 2009, MAPEDIR was implemented in 18 districts across eight states of India with the aim of addressing critical policy issues affecting maternal mortality. Based on this experience (the authors were closely associated with implementation of the process), we hypothesized that the implementation, utilization and up-scaling of the process were influenced by state-specific contextual factors that in turn affected MAPEDIR's ability to fulfill the related policy objectives. In this paper, based on the review of the initial experience in four districts across three states (Rajasthan, Madhya Pradesh and West Bengal), we examine this hypothesis. Based on the findings, we then propose an optimal model for introducing and scaling up a community-based maternal death inquiry and response process in India and in other similar settings.

## Methods

### Conceptual Framework

#### Maternal health policy objectives

Based on the vision, goals, objectives and guiding principles of the RCH-II programme, we identified the following national maternal health policy objectives that the MAPEDIR process could help achieve:

• Register all maternal deaths: whether MAPEDIR contributed to the un-hiding and investigation of maternal deaths;

• Evidence based planning: whether the information on causes and circumstances of maternal deaths generated by MAPEDIR led to the development of new interventions or strategies within the state plans;

• Decentralized Planning: whether the exercise led to the development and planning of district-specific interventions; and

• Community Participation: whether local communities participated in the development of new maternal health interventions based on the MAPEDIR-generated evidence.

#### Contextual factors

Several contextual factors were hypothesized to influence whether and to what extent the maternal death inquiry process will be effective in helping to meet the policy objectives. The choice of factors was based on brainstorming discussions among the study investigators on the potential reasons why the process will be more effective in some circumstances than in others. We further conducted a non-systematic literature review to identify the factors that define the performance of health systems. Some of the factors that emerged from the review were: leadership and accountability of health systems, staffing patterns for management and clinical functions, skill-mix of providers, adequate funding for health, responsiveness of services and management capacity [21-24]. Other factors, identified first by the brainstorming sessions, including social determinants such as women's status and social cohesiveness, and governance features such as responsiveness, were described by the literature as affecting both health outcomes and performance of health systems. The two sets of factors (generated from brainstorming and the literature review) were reviewed for their relevance in the context of the death inquiry process to arrive at the final set of factors. The factors were grouped as community, public health system and governance-related factors. For each of the identified factors, an indicator was derived to assess its status in the states.

#### Community factors

*Economic status*: Poorer communities have fewer resources to take effective actions on their own, and so are less able to overcome other negative contextual factors such as a weak civil society network, a non-responsive public health system or overly centralized government.

*Cohesiveness*: Communities that are more cohesive around class and caste are more likely to be more easily sensitized to maternal health issues and to launch effective and sustained actions for addressing maternal mortality. Inequities in the distribution of resources and services call into question the community's commitment to overcoming this problem.

*Status of women*: Lower status of women in the family and community will impede efforts to make the issue of maternal death more visible and to mobilize communities around maternal health. Therefore, while there is greater need for introducing the maternal death inquiry process precisely in those areas where women are accorded lower status, the process is likely to require a more intense and sustained effort.

*Civil society networks and influence*: Strong civil society networks are likely to take ownership for the maternal death inquiry process, or use the information and insights from such a process either to generate and strengthen community participatory action or enforce accountability on the public systems to deliver for women. The presence of such networks therefore is likely to accelerate implementation and up-scaling and enhance the effectiveness of such a process.

#### Public health systems factors

*Responsiveness and accountability*: Improving women's health requires taking and sustaining implementation of certain decisions such as ensuring the presence of staff at various levels of care skilled in providing emergency obstetric care. Wherever such a commitment exists, the maternal death inquiry process can guide prompt and appropriate action for addressing maternal mortality. In settings with less commitment, maternal death inquiry can serve as a tool in advocating for improved services and demanding increased accountability of the system.

*Capacity*: Conducting MAPEDIR requires some capacity in identifying and notifying maternal deaths, performing a sound inquiry, analyzing and interpreting data, sharing information, and identifying and implementing appropriate actions. The process is likely to be more effective where public health systems are stronger and possess the required capacity.

#### Governance factors

*Decentralization*: In territories with efficient and decentralized governance, local governments will play a stronger role in the maternal death inquiry process, which therefore will be more likely to be utilized for strengthening maternal health interventions and to be up-scaled faster.

*Responsiveness and accountability*: States that are more responsive to the healthcare needs of their population and direct resources to improving its health and welfare will support the implementation, utilization and up-scaling of maternal death inquiry and health interventions suggested by this process.

**Figure 2:**
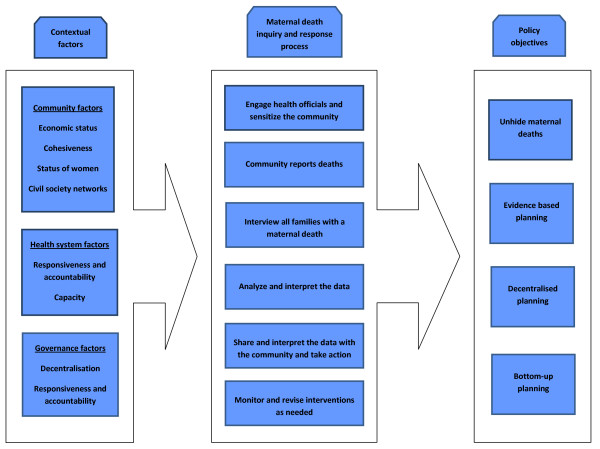
**Conceptual framework**.

We took a systems approach, rather than a linear approach, for describing the relationship between the contextual factors, the maternal death inquiry and response process, and the policy objectives (Figure 2). A contextual factor is likely to affect more than one step in the death inquiry and response process. For example, health systems capacity will influence not only analysis and interpretation of the data (step 4), but will also influence interviewing families (step 3), data sharing with communities (step 5), and monitoring and revising of interventions (step 6). Similarly, each step of the process is likely to influence achievement of more than one policy objective. For example, sharing and interpreting the data with the community will not only promote registering all maternal deaths, but will also advance community participation. Due to these multiple, potentially overlapping, influences, it is also possible that strengths in particular contextual factors may compensate for weaknesses in others, such as a strong NGO taking on data sharing in a setting where health system capacity for this task is low. In addition, factors may act together synergistically, as with higher status of women facilitating a community to take evidence-based action combined with NGO-guided advocacy advancing decentralized health planning linked to the community action.

## Analytic Approach

To examine the hypothesis, we took the following step-wise approach:

Step 1: Documented the contextual factors and case histories of MAPEDIR implementation and scaling-up across the three states;

Step 2: Examined the utilization of MAPEDIR and the degree to which it contributed to achievement of the policy objectives in the three states;

Step 3: We then scrutinized the case histories to assess the impact of the contextual factors in explaining the variation across the states in MAPEDIR's success in meeting the policy objectives.

Based on this assessment, we then considered how MAPEDIR might best be conducted in order to optimize its contribution to achieving the above stated policy objectives in India and other similar settings.

## Ethical Review

The MAPEDIR process was reviewed by the Johns Hopkins Committee for Human Research but was found to be exempt from its oversight due to being considered a programme intervention rather than a research study. However, high ethical standards were maintained by training the field personnel and programme managers in ethical aspects of human subjects research, administering and respecting informed consent to all death inquiry respondents, and by ensuring confidentiality of the respondent and the deceased in recording, storing and reporting the information.

## Results

### Contextual factors

#### Community factors

The populations of Rajasthan, Madhya Pradesh and West Bengal are rural and poor. Seventy-two percent to 77% of the states' 57-80 million residents live in tens of thousands of villages [25], high percentages of persons both rural and urban live below the official poverty line (BPL) (Table 1), and 62-85% of the poor inhabit the countryside [26]. Reflecting the high poverty rates, 30%, 36% and 29%, respectively, of the states' populace belong to a least privileged scheduled caste or tribe (SC/ST), compared to 24% nationally [25].

**Table 1:** Contextual factors hypothesized to affect MAPEDIR's effectiveness and indicators of their level

		State		
		
Contextual factor	Indicators	Rajasthan	Madhya Pradesh	West Bengal
*Community*				
Economic status	% population BPL (rural/urban) [26]	14.3/28.1	29.8/39.3	24.2/11.2
Community cohesiveness	% skilled birth attendance, by caste (SC/ST/other backward class/other)	27.0/23.9/37.3/42.8	25.7/13.9/32.6/49.5	46.2/24.5/64.5/44.5
Status of women	% female age 15-49 literacy	39.0	50.0	64.0
Civil society networks and influence	WSHGs: engagement in health activities; breadth of coverage*	Limited health activities (mostly involved in livelihood generation and micro-financing)	Assist with birth preparedness and arrange transport for normal or complicated labour; some districts	Promote MH and provide loans for medical emergencies; 300, 000+ WSHGs throughout WB
*Public health systems*				
Responsiveness and accountability	1. % CHCs with obstetrician posted	1. 32.2 (105/326)	1. 5.7 (13/229)	1. 43.2 (41/95)
	2. % women contacted in last 3 months by community health or nutrition worker	2. 11.7	2. 16.9	2. 23.3
Capacity	% blocks with a PH nurse*	0.0 (0/237 blocks)	0.0 (0/313 blocks)	> 80% (> 348/436 blocks)
*Governance*				
Decentralization	Decentralization index score [31]	0.35	0.59	0.75
Responsiveness and accountability	% health expenditure from public resources/out-of-pocket	30.0/70.0	16.6/83.4	21.6/78.4

BPL = below poverty line; CHC = community health centre; MH = maternal health; PH = public health; SC = scheduled castes; SHC = sub health centre; ST = scheduled tribes; WSHG = women's self-help group.

*UNICEF state health officers' personal communications.

^26^GOI Press Information Bureau: poverty estimates are based on household expenditure for essential items data collected by the National Sample Survey Organization of the Ministry of Statistics and Programme Implementation and state-specific poverty lines (rupees per capita per month): Rajasthan: 375/560; Madhya Pradesh: 328/570; West Bengal: 383/449.

^31^The initial decentralization measure is being revised, however, with expectation of little effect on the states' rankings (personal communication with Anoop Sadanandan).

While the limited resources of these communities may restrict their ability to act on their own, disparities in the treatment of these groups signals a low level of community cohesiveness that might further hamper MAPEDIR's implementation. Rural women are less than half as likely as their urban counterparts to be attended at delivery by a skilled person, but this discrepancy is evenly distributed across the three states, varying from 42-46% [27]. As might be expected, the poor, who more often reside in rural locales, suffer this same inequity, with scheduled tribes having the worst access to skilled birth attendance (Table 1). This gap in care is particularly glaring in Madhya Pradesh, where scheduled castes also have comparatively low access. In West Bengal, some backward castes actually have greater access to skilled birth attendance than more privileged groups [28]. As well, the status of women in West Bengal, as reflected in the female literacy rate, is the highest of the three states (Table 1). This compares to Madhya Pradesh and in particular to Rajasthan, where female literacy ranks far below India's overall 59% [27]. West Bengal's performance in these indicators may help explain its slowly narrowing rural-urban MMR gap [28,27].

Thousands of non-governmental organizations (NGO) operate in India. While objective measures of their effectiveness are lacking, health officials and others often form strong impressions of their credibility, which, along with their actual performance, affects their ability to function as partners in implementing MAPEDIR. Indigenous community groups, notably women's self-help groups, also are numerous throughout India and are another possible means of partnering with communities (Table 1). The many women's groups in West Bengal appear to offer an advantage for scaling up MAPEDIR.

#### Public health systems factors

West Bengal has the more responsive, accountable and capable public health system, outpacing Rajasthan and Madhya Pradesh in, for example, posting obstetricians at CHCs [29] and health workers in the community [27] (Table 1). Service quality at health facilities lags especially in Madhya Pradesh, though institutional deliveries increased from 41% in 2005-06 to 52% in 2006-07 following the introduction of pro-poor schemes like JSY. Nevertheless, West Bengal also has far to go to achieve its own stated targets such as posting an obstetrician at all 95 CHCs. This results in continued large pockets of poor service coverage such as in Purulia, the district chosen for initial MAPEDIR implementation.

#### Governance factors

By the indicators in Table 1 Rajasthan's health policy aims to mitigate inequities in the context of the state's poverty. While per capita income is the third lowest in India, nearly one third of health expenditure is from public resources, as compared to the national average of 20.6% [30]. Madhya Pradesh's public health expenditure is the lowest of the three states. However, its mid-range decentralization rank suggests some flexibility for government to take local initiatives [31]. As for West Bengal, its people are better informed and more politically conscious than those of most Indian states, and are quite vocal in demanding their rights. The state has a long history of responsive governance at all levels including the local Panchayat Raj Institutions (PRI). However, West Bengal's percent of health expenditure from public resources ranks midway between that of Madhya Pradesh and Rajasthan [30].

### MAPEDIR implementation (including key findings) and up-scaling

#### Implementation

Under the auspices of the RCH-II programme within the framework of the NRHM, MAPEDIR was piloted in Purulia district, West Bengal in May-June 2005 and maternal death inquiries began in July. Two blocks of Dholpur district, Rajasthan implemented maternal death inquiries in 2005-06 and the remainder of Dholpur started in 2007. Guna and Shivpuri districts, Madhya Pradesh began in early 2006.

Initially, low status of women in the family and community, poor awareness of maternal health issues and traditionally limited death notification impeded efforts in Rajasthan and Madhya Pradesh to raise the prominence of maternal death and mobilize communities to improve maternal care practices. In both states, as well, the public health system lacked the capacity to identify and investigate maternal deaths. Also, the PRI, including in the initial three MAPEDIR states, have traditionally taken a limited interest in health issues, as their development work most often has focused on construction projects.

These problems were addressed in Rajasthan by involving a local NGO to sensitize the community to maternal and perinatal health issues, including the need to notify and investigate all maternal deaths, and by training NGO personnel to conduct the death inquiries. In Madhya Pradesh, the districts partnered with UNICEF to train interviewers, including an NGO representative on each death inquiry team, and sensitized all village Panchayats on maternal health issues. Shivpuri district further enhanced death notification by engaging NGOs to sensitize communities, including village nutrition and health workers and volunteers.

In West Bengal, the Purulia district health authority took the lead role in conducting the death inquiries, engaging nutrition supervisors in helping to strengthen death reporting and interview families, and NGOs mainly in sharing the findings with local communities. This division of labor enabled Purulia to simultaneously implement MAPEDIR across the entire district, but exceeded the health authority's capacity to supervise the interview process, resulting in some loss in data quality. In all three states, UNICEF has supported analysis of the MAPEDIR data, and helped develop data entry and analysis software to ease transition of this function to district health offices.

##### Key findings

In all three states the death inquiries revealed that the deceased women were disproportionately from vulnerable groups (Table 2). In Dholpur, Guna/Shivpuri and Purulia, respectively, 49%, 55% and 61% of the women belonged to a scheduled caste or tribe, versus 25%, 31% and 37% of these same districts' populations [25]. Of particular importance in Purulia, where 41% of the women's families were BPL cardholders and 42% of villages are extremely isolated with poor road connections and no radio communication, was that no BPL cardholders knew this entitled them to free referral transport. Data mapping in Guna also showed that most deaths occurred in remote border villages lacking access to a skilled birth attendant.

**Table 2:** Selected MAPEDIR-collected maternal death indicators

	District, State		
	
Maternal health indicator	Dholpur, Rajasthan N = 35 n (%) or median (range)	Guna/Shivpuri, Madhya Pradesh N = 92 n (%) or median (range)	Purulia, West Bengal N = 102 n (%) or median (range)
Type of death (Ab, AN, LD [> 24 hours post delivery])	2, 6, 27 [14] (5.7, 17.1, 77.1, [40.0])	2, 1, 89 [28] (2.2, 1.0, 96.7 [30.4])	13 [7], 21, 68 [31] (12.7, 20.6, 66.7 [30.4])
Cause of death (H, S, E, Ob, A, O, U)*	10, 7, 6, 2, 5, 4, 1 (28.6, 20.0, 17.1, 5.7, 14.3, 11.4, 2.9)	27, 8, 25, 7, 9, 11, 5 (29.3, 8.7, 27.2, 7.6, 9.8, 12.0, 5.4)	23, 4, 19, 1, 9, 13, 33 (22.5, 3.9, 18.6, 1.0, 8.8, 12.7, 32.4)
Infant outcome in LD deaths: IUFD, stillbirth, born alive and died, alive	1, 12, 4, 10 (3.7, 44.4, 14.8, 37.0)	3, 17, 49, 20 (3.4, 19.1, 55.1, 22.5)	9, 11, 15, 33 (13.2, 16.2, 22.1, 48.5)
Age in years	26 (16-40)	25 (15-49)	24 (15-35)
Caste (SC/ST)	17 (48.6)	51 (55.4)	61/100 (61.0)
Kutcha house	15 (42.9)	66/92 (71.7)	83 (81.4)
Below Poverty Line card holder	11 (31.4)	58/91 (63.7)	39/95 (41.1)
Years of schooling	0 (1-10)	0 (0-12)	0 (0-10)
Age in years at marriage	16 (7-20)	17 (12-24)	17 (11-24)
Skilled birth attendance (LD deaths)	14/27 (51.9)	58/89 (65.2)	27/60 (45.0)
Sought formal care for the fatal illness: never, not first, first	15, 7, 13 (42.9, 20.0, 37.1)	28, 9, 52 (31.5, 10.1, 58.4)	17, 38, 47 (16.6, 37.3, 46.1)
Delay 1--time (hours) to decide to seek care	2 (0-32)	1 (0-15) (N = 44)	4 (0-216)
Delay 2a--time (hours) to arrange to seek care	2 (1-5) (N = 11)	0.5 (0.1-24) (N = 78)	1.0 (0-12.0)
Delay 2b--transport time (hours)	0.5 (0.5-2.0) (N = 8)	1 (0.2-3.0) (N = 82)	0.5 (0-4.2)
Reasons for not seeking formal care or not seeking formal care first: (perception, cost/transport, other) (multiple responses allowed)	4, 5, 9 (22.2, 27.8, 50.0) (18 reasons/18 women)	21, 19, 16 (37.5, 33.9, 28.6) (56 reasons/37 women)	24, 22, 6 (46.2, 42.3, 11.5) (52 reasons/34 women)
1^st ^action decision maker: (W, H, O)^†^ (% of all decision makers)	1, 9, 23 (3.0, 27.3, 69.7)	7, 41, 44 (7.6, 44.6, 47.8)	11, 68, 117^‡^ (5.6, 34.7, 59.7)
Referred from F1 to F2	11/17 (64.7)	42/61 (68.9)	48/67 (71.6)
Reasons for referral from F1 to F2 (complication, medicine, blood, procedure, specialist, equipment) (multiple responses allowed)	4, 1, 3, 0, 0, 3 (36.4, 9.0, 27.3, 0, 0, 27.3)	18, 14, 17, --, 23, 14 (20.9, 16.3, 19.8, --, 26.7, 16.3)	31, 6, 9, 23, 15, 8 (33.7, 6.5, 9.8, 25.0, 16.3, 8.7)
Out-of-pocket transport and treatment expenditure (rupees) at F1 and F2	1280 (150-13000) 2950 (350-11000)	300 (0-1500) 500 (0-3000)	800 (0-7100) 1860 (0-26500)

Ab = abortion; AN = antenatal; LD = during or after labor and delivery; IUFD = intrauterine fetal death; SC/ST = scheduled castes and tribes; F1 and F2 = first and second formal health care facilities visited for the fatal illness.

*H = hemorrhage, S = sepsis, E = eclampsia, Ob = obstructed labor, A = anemia, O = other, U = undetermined.

^†^W = the deceased woman, H = her husband, O = other.

^‡^Multiple decision makers allowed.

Careseeking problems were revealed by where women sought care and died and by delays. While more women in Purulia than in the other states sought formal care for their fatal complications, even there only 46% took this as their first action, with many first seeking care from an informal provider (Table 2). Of those who sought formal care, across the states on average it took 1 to 4 hours to decide this and another 0.5 to 2 hours to arrange transportation. The results were, for example, in Dholpur and Shivpuri, respectively, that 43% and 36% died at home and in Guna and Purulia, respectively, 16% and 12% died on route to the first facility visited. The interviews in all states also highlighted the reasons for delayed careseeking (Table 2).

Another problem identified was multiple referrals between facilities, with associated delays and costs. In all three states, about two-thirds of those who reached a facility were referred due to the facility's inability to provide the needed care. For example, in Dholpur, 10 women who required blood sought care at a health facility. Three of these women were referred for a lack of blood at the facility and only two of the 10 eventually were transfused. In Shivpuri, 10% of the women died while traveling between facilities. And in all states the average out-of-pocket costs were greater at the second than at the first facility, with some families spending many thousands of rupees (Table 2). This was a huge burden for the families, many of whom, for example 63% in Purulia, had to borrow money or sell assets just to hire a vehicle to go to the first facility. This also indicated a lack of preparedness on the part of the families for any eventuality associated with childbirth.

#### Up-scaling

In 2007 Rajasthan and Madhya Pradesh up-scaled MAPEDIR, respectively, to one and four additional districts, and Rajasthan and West Bengal mandated that all maternal deaths statewide be notified and investigated. As a result, in West Bengal 1292 of 1924 expected maternal deaths from April-October 2007 were reported and 903 investigated, albeit with a shortened version of the MAPEDIR death inquiry format and limited community participation. Rajasthan and Madhya Pradesh, respectively, allocated 3, 000, 000 and 300, 000 rupees to this effort in their yearly plans [32,33]. Rajasthan planned to up-scale further, initially setting up government-NGO death inquiry and advisory teams in four districts. Also, the obstetric helpline was replicated throughout Dholpur. State government's perception that NGOs working in the health sector have low capacity slowed wider up-scaling. In Madhya Pradesh, the up-scaling of MAPEDIR was overseen by the same safe motherhood committee that services all state maternal health interventions.

### MAPEDIR utilization and achievement of maternal health policy objectives

As a result of the MAPEDIR implementation efforts, large increases were seen in maternal death notification and especially in investigation (Table 3).

**Table 3:** Maternal health policies, objectives and indicators of MAPEDIR's effectiveness in helping to meet the objectives, and outcomes

		State (District) Outcomes		
	
National RCH policy	District maternal health policy objectives and indicators	Rajasthan (Dholpur) N (%)	Madhya Pradesh (Guna and Shivpuri) N (%)	West Bengal (Purulia) N (%)
Register all maternal deaths	*"Unhide" maternal deaths:* % of expected maternal deaths* reported by district before//after MAPEDIR implementation	Apr, 04-Mar, 05: 4% (5/116 deaths in 4 blocks)//Apr, 05-Mar, 06: 60% (35/58 deaths in 2 blocks)	Feb-Dec, 05: 47% (113/239)//Feb-Dec, 06: 57% (136/239)	July, 04-June, 05: 119% (114/96)//July, 05-June, 06: 127% (122/96)
Investigate all (or a sample of) reported maternal deaths	*Gather new evidence:* % of reported maternal deaths investigated by district before//after MAPEDIR implementation	Apr, 04-Mar, 05: 0% (0/5)//Apr, 05-Mar, 06: 100% (35/35)	Feb-Dec, 05: 0% (0/113)//Feb-Dec, 06: 100% (136/136)	July, 04-June, 05: 0% (0/114)//July, 05-June, 06: 86% (105/122)
Implement RCH programme planning and management: • Evidence-based	*The state uses the new evidence:* New maternal health interventions developed by/with the state health system based on MAPEDIR data	Statewide obstetric help line (implementation delayed); 141 FRU blood storage units equipped	None	Made all public maternity beds non-paying; expanded JSY to all SC/ST and BPL women; implemented new rural referral transport system
• Decentralized	*Decentralized MH planning:* New maternal health interventions initiated at district level based on MAPEDIR data	District health society planned and mobilized obstetric helpline and referral transport system by partnering with civil society	Guna: mapped maternal deaths to prioritize & upgrade remote SHCs for 24 × 7 safe delivery services; Guna and Shivpuri: ensured 24 × 7 referral transport to all PHCs via call center and secured vehicles	None
• Bottom-up	*Community participation:* New maternal health interventions developed by or with communities based on MAPEDIR data	Taxi union & NGO collaborated with district in implementing and running the obstetric help line and referral transport system	Guna: block PRI ensured referral transport for remote villages to upgraded SHC; Guna: communities donated 6 of 22 referral vehicles	GP-initiated 8 van rickshaws in 4 remote GPs of 4 Purulia blocks

BPL = below poverty line; FRU = first referral unit; GP = gram panchayat (local governance board); JSY = Janani Suraksha Yojana (institutional care incentive scheme); MH = maternal health; NGO = non-governmental organization; PHC = primary health centre; PRI = Panchayat Raj Institutions (local governance system); RCH = Reproductive and Child Health Programme; SC/ST = scheduled castes and tribes; SHC = sub health centre.

*Expected deaths = District (Block) population*State crude birth rate*State MMR (from 2001-03 Sample Registration System Special Survey of Deaths): Dholpur: 1, 000, 000 (2 blocks = 500, 000)*26/1000*445/100, 000; Guna/Shivpuri: 2, 600, 000*26.5/1000*379/100, 000*(11/12); Purulia: 2, 700, 000*18.4/1000*194/100, 000.

#### Data sharing and use

The MAPEDIR data were shared at multiple levels in all states. Thousands of villagers attended meetings in Dholpur, Rajasthan and jansunwais (public hearings) were convened at district and state headquarters to discuss the findings of the death inquiries. Follow-up multi-sector government meetings were held to develop an action plan based on the findings. In Madhya Pradesh, the data were shared with the state government and civil society network, at monthly district-block mixed civic-health authority task force meetings, and with the community via the PRI system. NGO representatives participating in monthly meetings in Shivpuri also took the messages to the community via local NGOs. In Purulia, the PRI took an interest when NGOs began sharing the interview findings at Gram Panchayat (multi-village) meetings, involving Panchayat functionaries, health and nutrition workers, and local community representatives. The data also were shared with the Purulia and West Bengal health authorities. Later, two of the three states and most districts began using MAPEDIR data in developing their RCH-II programme implementation plans (PIP).

#### Response

These efforts culminated in achievements that are reflected in the indicators of MAPEDIR's effectiveness as a policy implementation tool, with local communities and districts responding to the death inquiry findings in various and creative ways (Table 3). In Dholpur, where it took 2 hours on average to arrange to seek health care and women often first went to facilities ill-equipped to help them, the district set up an obstetric helpline in Bari block in collaboration with the local NGO and taxi union. UNICEF provided technical support and the programme linked with the national JSY to assure financial viability. From January 2006-July 2007, the service transported almost 1400 women. Moreover, while deliveries at the Bari CHC increased by 1.4 times in 2006 over 2005, C-sections increased 15 fold with the same staffing levels. Observation suggests that this increase was due to greater demand for quality services by families assisted by the NGO. The state responded to the MAPEDIR findings by equipping 141 FRUs with a blood storage unit.

In response to the mapping of maternal deaths in Guna, with UNICEF support the district upgraded a sub-centre in an underserved border area for 24 × 7 safe deliveries. The local Gram Panchayat identified willing vehicle owners and coordinated emergency transport to the sub-centre for women in remote villages. The success of this effort led Guna district to revitalize additional facilities in remote areas to conduct safe deliveries. In addition, through a public-private-NGO partnership with UNICEF technical support and monitoring by state officials, the district arranged for a call center and free ambulance transport to 22 facilities providing 24 × 7 deliveries. The service transported 1153 patients in the first 6 months, and was then extended to Shivpuri.

In Purulia, with a high percentage of isolated villages, sharing of the data on maternal deaths led several pradhans (local PRI leaders) to initiate and fund an emergency referral transport system consisting of locally appropriate "van rickshaws." Women's self-help groups managed the system, which interlinked difficult-to-reach areas with main roads. By December 2007, with persistent communication by NGO, PRI and self-help group members, 28 pregnant women and many other patients of five Gram Panchayats had utilized the service.

The state governments of Rajasthan and West Bengal also implemented new maternal health interventions based partly or wholly on the MAPEDIR findings (Table 3).

## Discussion

Despite an abundance of resources and a history of implementing maternal health initiatives, India has lagged behind in meeting its own maternal mortality reduction objectives. The first hurdle has been bringing the problem to light. The full scale of the deaths has remained uncounted. A village may experience one maternal death every few years, decreasing the urgency to act, and inadequately trained and resourced health workers covering multiple villages have felt discouraged to report deaths for which they might be blamed. With RCH-II and NRHM, India is making a push to overcome past obstacles, to highlight maternal mortality as a priority issue, bring resources to bear on pivotal well defined high impact strategies, and strengthen local implementation by engaging communities as active participants and partners.

MAPEDIR was developed to help address these critical maternal health policy issues by synergizing simultaneous action on both sides of the quality RCH services supply-demand equation. Facility- and community-based maternal death audits and reviews have been utilized in other developing countries as a means of identifying and focusing action on social and health care quality issues affecting maternal survival [17-20]. In the high mortality states of India, where most births and many maternal deaths occur at home, MAPEDIR was launched at the community level. In the three initial states, it was felt to be important to examine how local contextual factors have affected the success of MAPEDIR in meeting critical maternal health policy objectives, and to consider how this might influence the design of an appropriate MAPEDIR implementation strategy.

### Unhide maternal deaths and gather new evidence

MAPEDIR led to increases in maternal death notification and investigation whether civil society or government took charge of these tasks. In Rajasthan, where the low capacity public health system was unable to fulfill these functions, it was only the presence of a strong NGO that enabled MAPEDIR to start, this despite the general weakness of civil society in Rajasthan. In addition to conducting death inquiries, the NGO overcame the barriers of low cohesiveness and women's low social status to the reporting of maternal deaths by sensitizing the community about maternal and perinatal health. Madhya Pradesh, with both a weak public health system and variable NGO credibility, strategically partnered with UNICEF and selected NGOs to undertake the death inquiries. The moderate strength of women's status in Madhya Pradesh, as indicated by its female literacy score, may have facilitated NGO efforts in Shivpuri to increase community death reporting. This factor also may have allowed Guna to accomplish the same via its more active governance and stronger public health response, despite the state's relatively low public health ranking. West Bengal had the advantages of its general strength in all three major categories of contextual factors, and was able to enlist a reasonably credible NGO in Purulia to sensitize the community. The main challenge in this setting was for the responsive and capable public health system to incorporate the new task of conducting death inquiries into the regular public health supervisory system. The outcome was a small increase in maternal death reporting beyond the historically high levels, combined with, as in the other states, a large boost in death investigation.

### Bottom-up maternal health planning

NGO involvement was the cornerstone of community participation in Rajasthan and played a facilitating role in West Bengal, with maternal deaths being vigorously discussed and appropriate local interventions developed. While, in Rajasthan, the NGO also played a key role in running the obstetric helpline, in West Bengal the van rickshaws were deployed by strong local government working with indigenous self-help groups. NGOs were minimally involved in Guna, Madhya Pradesh, yet MAPEDIR became the impetus for regular grassroots health audits at which the maternal death data were reviewed. The resulting intervention, community boosting of emergency transport to a sub-centre upgraded by the district to provide 24 × 7 skilled delivery services in response to the same data, was taken district-wide by the government with community participation and support by development partners. This was perhaps as close as any of the MAPEDIR-activated initiatives came to fulfilling the RCH-II priority of community participation in evidence-based health planning. It is instructive to note that this occurred by implementing MAPEDIR through the PRI system, historically disinterested in women's health, but where local governance and community participation closely meld and are uniquely situated to unite MAPEDIR's two means of action for empowered communities--advocacy for quality services and development of local interventions --into the ideal demand-supply prescription. This approach took advantage of the two contextual factors of moderate strength in Madhya Pradesh, i.e., its decentralization index score and women's status in the community.

### Evidence-based and decentralized maternal health planning

Health system participation in evidence-based maternal health interventions was enabled mainly by responsive and accountable governance, rather than actions taken by the health system itself, but sometimes this resulted in a top-down approach poorly coordinated with community action. State government took the lead in West Bengal in developing a referral transport scheme and the local PRI system complemented this with the van rickshaw plan in Purulia. Yet, opportunities for forging links between the levels, such as ensuring timely contact between van rickshaws and ambulances, were missed and the intervention was not taken district wide. This may indicate some over decentralization of local government in West Bengal, rather than finding the proper balance between encouraging local initiative and up-scaling to increase coverage. In Rajasthan, district government overcame its weakness by partnering with civil society and UNICEF in developing the obstetric helpline. The state public health system equipped blood storage units in response to the MAPEDIR findings but, as predicted by its low responsiveness and capacity ratings, was unable to resolve staffing shortages needed to skillfully use the blood. In Madhya Pradesh, state government monitoring of MAPEDIR-related activities and positive feedback to Guna and Shivpuri for their innovative work were important motivators for the actions taken by the district public health systems. This was the one instance in which a contextual factor's rating (low responsiveness and accountability) did not accurately predict MAPEDIR's effect (strong support for evidence-based RCH planning and management) at the state level.

All three states found MAPEDIR helpful enough in meeting maternal health policy objectives to decide to up-scale the activity using government funding. It is too early to say which contextual factors influenced whether MAPEDIR was successfully up-scaled or not, but based on the experience to-date it is evident that this decision must be taken by top leaders of the state's health administration and so depends on MAPEDIR also raising awareness and the profile of maternal health and mortality among the country's highest political echelon. Thus, by accomplishing its first objective of unhiding maternal deaths can MAPEDIR succeed in continuing to raise awareness and empower local communities and government with the evidence needed to decrease maternal mortality.

Some limitations to the analysis warrant discussion. While the contextual factors closely followed their hypothesized influence on MAPEDIR's success in helping to meet the policy objectives, some factors' effects were not fully predictable, partly due to their state rankings not always holding true at the district level, and because strengths in some factors were able to overcome other weak ones. It should also be kept in mind that this analysis has led to identifying a set of factors that appear to determine the influence of a community-based maternal death enquiry process on achieving certain policy objectives. Further work would be required to validate the association. The implications of these finding were taken into account in developing the proposed model discussed below.

Also, it cannot be categorically stated that MAPEDIR was the only factor that led to each maternal health policy outcome described in this paper. MAPEDIR was implemented in a programmatic, not a research, mode, so it was not possible to conduct a cluster randomized controlled trial; nor did we assess comparison areas where MAPEDIR was not being implemented. However, there were indisputably solo links between the MAPEDIR process and several outcomes at the block and district levels, such as development of the van rickshaw intervention in Purulia, the obstetric help line in Dholpur, and the upgrading of remote SHCs in Guna based on the mapping of maternal deaths. MAPEDIR-conceived interventions sometimes interacted synergistically with other activities, such as the Dholpur help line's linking with the national JSY program to assure financial viability. We did not prove that the help line increased institutional deliveries beyond what JSY would have achieved by itself, but even if not, it is highly likely that the quality of care was positively influenced by the help line's advocacy for women, as shown by the 15-fold increase in C-sections accompanying the 1.4 times increase in institutional deliveries. Statewide interventions such as the expansion of the JSY program in West Bengal to all SC/ST and BPL women were influenced by other factors as well, but even in such cases the evidence from the death inquiries often played a catalytic role, tipping the balance between discussion and action. Lastly, even though a before/after analysis without a separate comparison area does not provide definitive scientific evidence, it is difficult to explain the often dramatic increases in maternal death reporting and investigation with MAPEDIR other than as a direct result of the MAPEDIR process.

## Conclusion and proposed model

The findings supported the hypothesis that contextual factors strongly affect the success of a maternal death inquiry and response process in helping to meet critical maternal health policy objectives. Strong civil society inputs, in particular, were essential in enabling poorly cohesive communities with low women's status to participate in MAPEDIR, and also helped fill the gaps left by weak public health systems and government and complemented these when they were stronger. The case of Guna, in Madhya Pradesh, however, showed that MAPEDIR can work well even in the poorest of settings with minimal civil society inputs when the local government and health system are responsive and accountable and there is a supportive development partner. Good governance facilitated health system participation in MAPEDIR, but did not ensure the capacity to manage the core MAPEDIR functions--data collection, analysis and interpretation in partnership with communities to develop evidence-based, highly effective RCH interventions. Analyzing the contextual factors' contributions to the successes and shortcomings of the initial MAPEDIR efforts suggests a model for optimizing maternal death inquiry implementation and utilization in India and other similar developing country settings. In applying the model, whenever possible, sites should assess the strengths and weaknesses of contextual factors at the district level, rather than the state or provincial level, as these will often be more decisive in contributing to the process' success. Strengths in some factors that can overcome weaknesses in others should be sought. The optimal model includes the need for flexibility.

First, MAPEDIR must be mandated by the district governments, and given ongoing support as the means by which certain critical maternal health policy objectives will be achieved. At its core, MAPEDIR is an advanced health programme planning tool. Hence, coordination of the various MAPEDIR functions should be housed in the district health office or, within India's RCH programme structure, in the district NRHM office. Especially in communities that are weakly cohesive or where the status of women is low, the district should consider contracting with or otherwise engaging a strong NGO to assist it in partnering with the community to strengthen sensitization to maternal health issues and the need for maternal death notification. The health administration often will want to supervise and have its own workers conduct the death inquiries. However, given the many demands on health workers' time, confidentiality concerns, and that some families may blame the system for the woman's death, this task may be better served by a partner NGO.

The district office should take on the core MAPEDIR functions of data management, analysis and interpretation as part of its ongoing surveillance programme. A development partner may need to provide technical assistance to strengthen the office's capacity in these areas. The office should organize a highly participatory and consultative process to share and interpret the findings and develop evidence-based interventions in partnership with the community, again, if necessary, with NGO assistance and development partner support. Data sharing and interpretation should take place at all levels, from the village, where a participatory learning approach will facilitate empowerment and action, to village clusters with communal ties, and up the scale of local sub-district divisions. The district office should also engage local health offices in data sharing and interpretation workshops, which play a vital role in sensitizing providers to the circumstances of the women that contributed to their death and promote partnering with the community in finding local solutions. All data sharing must be conducted in a non-blaming atmosphere and with full protection of confidentiality, both of the deceased women and their families and any involved health workers. This will encourage openness and a search for solutions to problems affecting the community and the health system as a whole, which more often contribute to a woman's death than the actions of any particular individual.

Where possible, these activities should be integrated into the normal operations of a multi-tiered, multi-sectoral local governance system such as India's PRI. This will foster their institutionalization at multiple levels where the MAPEDIR findings are regularly discussed as a health monitoring and programming tool. The health office should coordinate and support decisions taken at the various levels to assure that any new interventions are integrated across the levels to provide for their optimal impact. State actions should be guided by the MAPEDIR findings and interventions developed by districts, either by up-scaling to additional districts or implementing complementary statewide programmes. The state should exercise oversight of district activities, keeping the spotlight on exemplary programmes as a motivator for local initiative and an example for others.

Following this model should optimize the up-scaling of MAPEDIR to additional districts and states, fully integrated into the health programme's usual management structure, rather than as a short-term project. MAPEDIR is an awareness-raising, empowering, decision-making tool that can adapt to new environments and, as it has in the Indian states examined in this paper, itself become an important contextual factor in the battle against maternal mortality.

## Competing interests

HDK, PM, NG, SB and GA provided technical assistance to the states in implementing MAPEDIR, HDK as a consultant to UNICEF and the others as UNICEF staff members. MB headed the UNICEF health section that provided this assistance. ABB conducted a training programme for MAPEDIR field workers in West Bengal. AM is with the Government of Madhya Pradesh health programme that implemented MAPEDIR. The authors have no financial or other competing interests.

## Authors' contributions

HDK, PM and MB conceived of the study and its design. HDK drafted the manuscript, with help from PM, NG, SB and GA. PM and NG provided data for tables 1 and 3. AM, ABB, SB, NG, PM and HDK contributed to acquisition of the primary data in table 2. HDK, NG, PM and ABB analyzed and interpreted the primary data in table 2. All the authors critically reviewed and approved the final manuscript.
